# *Fusobacterium nucleatum*: a keystone bridging pathogen in polymicrobial biofilms and host immune dysregulation

**DOI:** 10.3389/fcimb.2026.1981479

**Published:** 2026-10-01

**Authors:** Jinwen Sima, Siyu Ji, Guanbao Zhang, Suling Li, Siyu Sun, Wangping Dong, Qiancheng Luo, Qingju Bian, Shuyi Cheng, Yunshuai Wang, Xiaofei Cheng

**Affiliations:** 1 North Henan Medical University, Xinxiang, China; 2 Henan Medical University, Xinxiang, China; 3 Zhejiang Chinese Medical University, Hangzhou, China; 4 Zhengzhou Central Hospital, Zhengzhou, China

**Keywords:** colorectal cancer, *Fusobacterium nucleatum*, immune dysregulation, periodontitis, polymicrobial biofilms

## Abstract

Oral infectious diseases such as periodontitis stem from disrupted homeostasis between host immunity and polymicrobial biofilm communities. Fusobacterium nucleatum, a ubiquitous oral commensal, is a key intermediate colonizer with well-documented interspecies coaggregation activity. It has been proposed as a bridging pathobiont that contributes to microbial dysbiosis and immune dysregulation at the host–microbe interface, though its *in vivo* structural bridging function remains debated. This mini-review synthesizes recent advances in understanding the dual functions of F. nucleatum in interspecies microbial interactions and host pathogenic crosstalk. We first delineate the molecular basis of its structural bridging role, focusing on surface adhesins FadA and Fap2 that mediate interspecies coaggregation, support colonization by late-periodontopathogens such as Porphyromonas gingivalis, and enable epithelial barrier invasion. We then dissect mechanisms by which F. nucleatum modulates innate and adaptive immune responses, propagating chronic inflammatory tissue damage while subverting host clearance pathways. Furthermore, we discuss synergistic pathogenicity with co-occurring pathogens and the systemic implications of disseminated F. nucleatum in extraoral disorders including colorectal cancer and adverse pregnancy outcomes. Finally, we highlight the translational promise of targeting F. nucleatum-mediated adhesion and immunomodulation as an ecological therapeutic strategy for oral infections and their systemic sequelae.

## 1 Introduction

Chronic oral infections such as periodontitis arise from disrupted homeostasis between host mucosal immunity and polymicrobial biofilms in subgingival niche (Darveau, 2010). Disease pathogenesis is not driven by single high-virulence pathogens, but by community-level dysbiosis: shifts in microbial composition, metabolic crosstalk, and interbacterial cooperation remodel local microenvironments toward persistent inflammation (Hajishengallis and Lamont, 2012). Oral dysbiosis can extend beyond the oral cavity; mucosal barrier impairment enables bacterial translocation, which fuels low-grade systemic inflammation and modifies risk for distant organ diseases (Peng et al., 2022).

Fusobacterium nucleatum, an obligate anaerobic Gram-negative bacterium, is a ubiquitous commensal in dental biofilms of both healthy and periodontitis subjects (Yang et al., 2025). It has been widely studied as a candidate keystone bridging pathobiont that may drive biofilm maturation and mediate pathological host-microbe interactions (Jiang et al., 2026), though *in vivo* evidence for its structural bridging role remains incomplete and subject to ongoing debate. Unique among oral anaerobes, F. nucleatum exhibits broad interspecies coaggregation capacity, linking early plaque colonizers and late periodontopathogens such as Porphyromonas gingivalis (Yamaguchi-Kuroda et al., 2023). This property allows it to act as a structural scaffold for pathogenic multispecies consortia, while its surface virulence factors mediate epithelial adhesion, invasion, and modulation of innate and adaptive immunity (Meng et al., 2021).

Existing reviews focus mainly on either its oral biofilm ecology or colorectal oncogenic roles, with few systematically integrating its dual function as a microbial bridge and immune modulator at the host-microbe interface (Li et al., 2025). This mini-review synthesizes recent mechanistic advances in F. nucleatum-driven polymicrobial dysbiosis and immune dysregulation. We delineate the molecular basis of interbacterial bridging and epithelial barrier penetration, dissect its roles in inflammatory tissue injury and immune evasion, and summarize synergistic pathogenicity with co-occurring oral bacteria. We also address systemic consequences of extraoral dissemination, including adverse pregnancy outcomes and colorectal cancer, and discuss translational strategies targeting F. nucleatum adhesion and immunomodulation.

## 2 Microbial bridging and epithelial invasion by *F. nucleatum*

### 2.1 Ecological niche in sequential biofilm assembly

Oral dental biofilms develop through a highly ordered sequential colonization process in classical stepwise models, which are predominantly established based on *in vitro* biofilm reconstitution studies. Early colonizers, predominantly streptococci and actinomycetes, first adhere to the salivary pellicle on tooth surfaces to form the foundational biofilm layer. Intermediate bridging species then integrate into the initial biofilm structure, creating attachment niches for late-arriving periodontopathogens such as Porphyromonas gingivalis and Tannerella forsythia (Zhang et al., 2022).

Fusobacterium nucleatum is a dominant intermediate colonizer in subgingival biofilms, with well-characterized broad-spectrum interspecies coaggregation activity *in vitro*. This unique property has given rise to the longstanding hypothesis that it functions as a structural node driving the transition from a commensal community to a pathogenic consortium. In support of its functional role in biofilm maturation, *in vitro* co-culture experiments have demonstrated that F. nucleatum can enhance the growth of the motile periodontopathogen Treponema denticola via non-contact, diffusible factor-mediated mechanisms, which may further promote pathogen enrichment during later stages of biofilm development (Kikuchi et al., 2025). Consistent with these *in vitro* findings, clinical cross-sectional data show that F. nucleatum abundance increases markedly with progression from gingival health to periodontitis, and its subgingival levels are positively correlated with enrichment of late periodontopathogens in plaque samples (Griffen et al., 2012).

However, the *in vivo* physiological relevance of the classical bridging model remains debated, and two key lines of evidence challenge the notion that F. nucleatum is an obligate bridging species for periodontal biofilm assembly. First, ex vivo studies have demonstrated that aggregates of primary colonizing bacteria can pre-assemble in saliva prior to adhesion to the tooth surface, bypassing the need for stepwise, surface-mediated interspecies bridging (Simon-Soro et al., 2022). Second, direct physical adherence interactions between late periodontopathogens and early colonizers have been well documented at the molecular level; for example, P. gingivalis can bind directly to Streptococcus gordonii via its surface adhesin Mfa1, indicating that intermediate bridging species are not strictly required for late pathogen colonization (Park et al., 2005).

### 2.2 Surface adhesins mediating interspecies coaggregation

The broad interspecies coaggregation capacity of F. nucleatum observed *in vitro* is mediated by a panel of surface-expressed adhesins with distinct ligand specificities, which constitutes the molecular basis underlying its proposed bridging role in biofilm assembly. These adhesins operate in a division-of-labor manner, mediating binding to early colonizers, late periodontopathogens, and host epithelial cells respectively.

FadA is a well-characterized multifunctional adhesin involved in both interbacterial coaggregation and host cell interaction. It exists in two isoforms: membrane-bound FadA directly mediates physical binding to Gram-positive early colonizers, while secreted FadA assembles into amyloid-like fibrils that reinforce the structural integrity of mixed-species biofilms and enhance overall bacterial adherence efficiency (Britton et al., 2025). Beyond interbacterial interactions, FadA serves as a key virulence factor that enables F. nucleatum to adhere to and invade oral epithelial cells, functionally linking its biofilm-associated role to mucosal barrier penetration.

Fap2 is a galactose-specific adhesin that primarily mediates coaggregation with late Gram-negative periodontopathogens, most notably P. gingivalis. It recognizes galactose-containing glycan moieties on the surface of partner bacteria, thereby stabilizing the spatial architecture of polymicrobial biofilms (G C B and Wu, 2025). Importantly, this interspecies interaction is subject to dynamic regulation: diffusible signaling molecules released by P. gingivalis can upregulate Fap2 expression in F. nucleatum, which in turn further augments mixed biofilm maturation *in vitro* (Yamaguchi-Kuroda et al., 2023).

As an auxiliary adhesin, RadD complements the above functions by mediating specific coaggregation with oral streptococci. It is an arginine-inhibitable outer membrane protein that strengthens the physical connection between F. nucleatum and early-colonizing Gram-positive species (Sakanaka et al., 2022). The coordinated action of FadA, Fap2 and RadD collectively establishes F. nucleatum as a central interaction hub in reconstituted *in vitro* multispecies biofilm systems (Kaplan et al., 2009). Nevertheless, whether these adhesin-mediated bridging effects play an equally indispensable role in native, structurally complex subgingival plaques *in vivo* remains to be validated by high-resolution *in situ* imaging studies.

### 2.3 Epithelial barrier penetration

Beyond structuring microbial communities, F. nucleatum adhesins mediate direct interactions with oral epithelial cells, facilitating mucosal barrier penetration. FadA binds E-cadherin on epithelial cell surfaces, triggering cytoskeletal rearrangements that promote bacterial internalization (Han et al., 2005). This adhesion process also disrupts tight junction integrity, increasing epithelial permeability and creating routes for deeper tissue invasion by F. nucleatum and associated pathogens (Ellett et al., 2023). Epithelial barrier breach is a critical step linking oral biofilm dysbiosis to local tissue damage and systemic bacterial dissemination. The invasive capacity of F. nucleatum not only sustains local chronic infection but also enables bacterial translocation into the bloodstream, representing a mechanistic connection between oral infection and extraoral diseases (Farrugia et al., 2022). The dual functions of F. nucleatum — acting as a structural hub for polymicrobial biofilm assembly and as an invasive driver of epithelial barrier disruption — collectively constitute the core structural basis for the initiation of oral dysbiosis and subsequent bacterial tissue invasion. The underlying molecular and cellular mechanisms are schematically illustrated in **Figure 1**.

**Figure 1:**
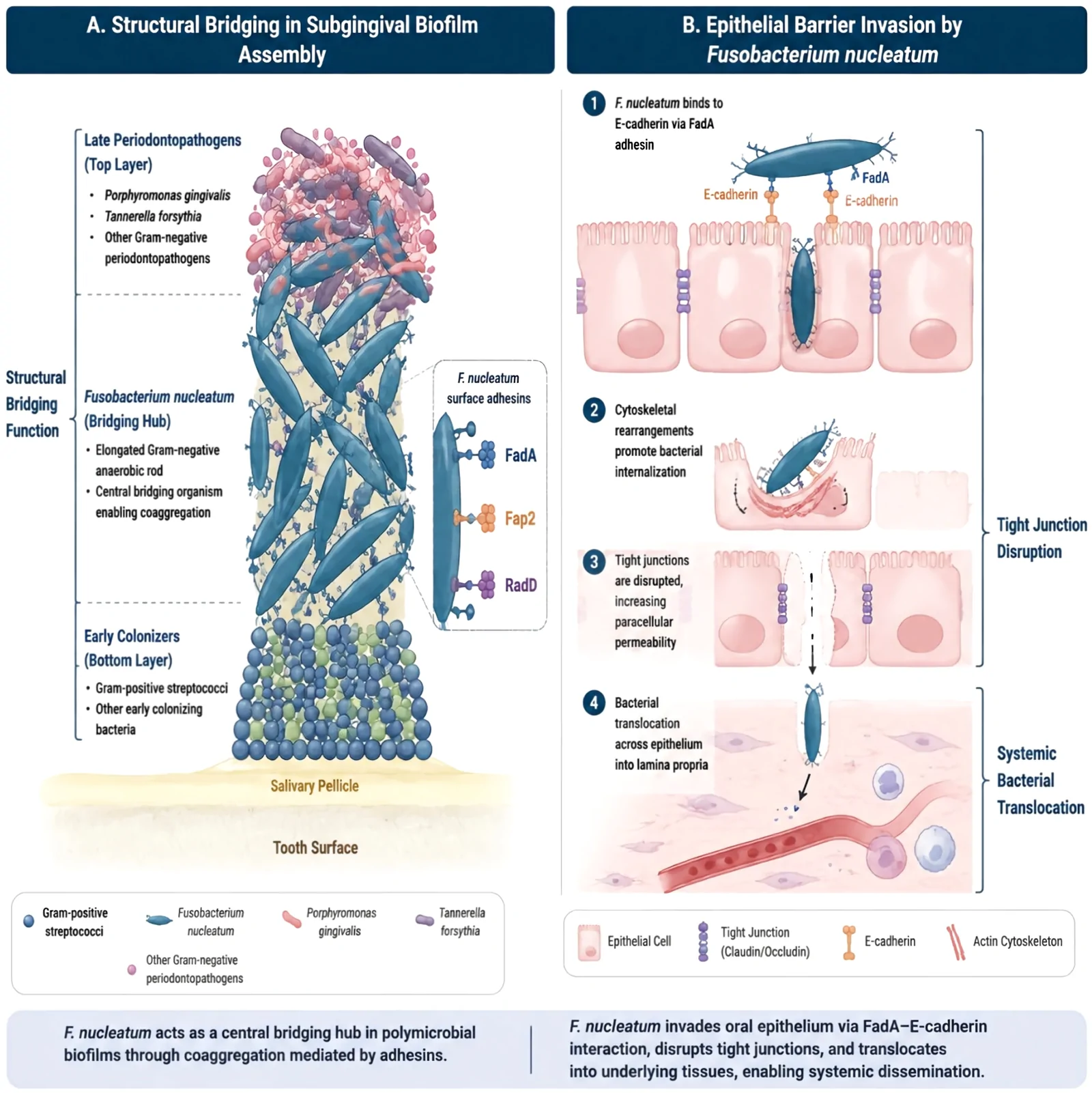
Mechanisms of microbial bridging and epithelial barrier invasion mediated by *Fusobacterium nucleatum.*(Schematic representation of the dual roles of Fusobacterium nucleatum in subgingival biofilm maturation and oral epithelial barrier penetration. **(A)** Structural bridging function in subgingival polymicrobial biofilm assembly. F. nucleatum serves as the central intermediate bridging organism, linking early Gram-positive colonizers (e.g., oral streptococci) and late Gram-negative periodontopathogens (e.g., Porphyromonas gingivalis, Tannerella forsythia) via three key surface adhesins (FadA, Fap2, RadD), thereby supporting the sequential maturation of pathogenic biofilm consortia on the salivary pellicle of the tooth surface. **(B)** Epithelial barrier invasion and bacterial translocation. F. nucleatum binds to E-cadherin on oral epithelial cells via its surface adhesin FadA, which triggers intracellular cytoskeletal rearrangements and promotes bacterial internalization. This interaction further disrupts tight junction integrity, increases paracellular permeability, and enables bacterial translocation across the epithelial layer into the underlying lamina propria and systemic circulation.).

## 3 Immune modulation and systemic pathogenic effects of *F. nucleatum*

### 3.1 Activation of local innate immunity and inflammatory cascade amplification

After breaching the oral epithelial barrier, F. nucleatum invades the subepithelial lamina propria and interacts with resident innate immune cells, triggering a robust inflammatory cascade that drives local tissue damage. Its surface pathogen-associated molecular patterns, including lipopolysaccharide and multiple outer membrane proteins, are recognized by Toll-like receptor 2 and Toll-like receptor 4 on macrophages and dendritic cells. This recognition activates intracellular NF-κB signaling pathways and the NLRP3 inflammasome, inducing the transcription and secretion of key pro-inflammatory cytokines such as tumor necrosis factor-α, interleukin-1β, and interleukin-6 (Chen et al., 2022). These cytokines further recruit circulating neutrophils and monocytes to the lesion site, amplify the inflammatory response, and activate matrix metalloproteinases and osteoclasts, which directly mediate periodontal connective tissue degradation and alveolar bone resorption, the core pathological features of progressive periodontitis.

### 3.2 Immune evasion mechanisms and maintenance of chronic infection

Despite inducing strong inflammatory responses, F. nucleatum has evolved multiple sophisticated strategies to evade host immune clearance and establish persistent chronic infection. It disrupts neutrophil-mediated antimicrobial defense by interfering with phagosome maturation and suppressing the production of reactive oxygen species, thereby reducing intracellular killing efficiency and allowing bacterial survival inside phagocytes (Kurgan et al., 2017). In addition, F. nucleatum drives the recruitment and accumulation of myeloid-derived suppressor cells in periodontal lesions. These cells exert potent immunosuppressive effects by inhibiting the proliferation and effector functions of CD4+ and CD8+ T lymphocytes, blunting adaptive immune surveillance and preventing effective eradication of the pathogen (Liang et al., 2022). The combination of sustained inflammatory activation and impaired immune clearance creates a vicious cycle that perpetuates chronic mucosal inflammation and progressive tissue destruction.

### 3.3 Systemic dissemination and pathogenic effects on distant organs

The invasive property of F. nucleatum enables it to penetrate deeper tissues and enter the systemic circulation, leading to hematogenous dissemination to distant organs and contributing to the pathogenesis of various extraoral diseases. It adheres to vascular endothelial cells via conserved surface adhesins, disrupts endothelial barrier integrity, and mediates transendothelial migration to access underlying parenchymal tissues, which is a prerequisite for its colonization at secondary sites (Fardini et al., 2011). In tumor microenvironments, F. nucleatum selectively accumulates and reshapes the immune landscape to suppress anti-tumor immunity, while also directly activating pro-tumorigenic signaling pathways in cancer cells to promote proliferation, invasion, and chemoresistance (Yu et al., 2017). Beyond oncology, systemic spread of F. nucleatum has been implicated in adverse pregnancy outcomes, atherosclerotic cardiovascular inflammation, and neurodegenerative disorders, where it exerts pathogenic effects through conserved immunomodulatory mechanisms, demonstrating its broad impact on human health beyond the oral cavity (Fan et al., 2022).

The aforementioned immune regulatory and pathogenic processes involve multiple molecular pathways and heterogeneous disease phenotypes. To systematically clarify the corresponding relationships between functional mechanisms, cellular targets and clinical manifestations, the core immune functions and systemic pathogenic profiles of F. nucleatum are summarized in **Table 1**.

**Table 1:** Summary of immune regulatory mechanisms and systemic pathogenic effects of *Fusobacterium nucleatum*.

Functional module	Primary target cells	Key molecular & cellular mechanisms	Core biological effects	Associated diseases
Innate immune activation & inflammatory cascade amplification	Macrophages, dendritic cells, neutrophils, osteoclasts	TLR2/TLR4 recognition of surface PAMPs; NF-κB and NLRP3 inflammasome activation; secretion of TNF-α, IL-1β and IL-6	Innate immune cell recruitment; matrix metalloproteinase induction; alveolar bone resorption	Chronic periodontitis; alveolar bone loss; local mucosal inflammation
Immune evasion & chronic infection maintenance	Neutrophils, CD4+/CD8+ T lymphocytes, myeloid-derived suppressor cells	Inhibition of phagosome maturation and ROS production; MDSC recruitment; suppression of T cell proliferation and effector function	Impaired bacterial clearance; sustained inflammatory state; escape from adaptive immune surveillance	Persistent periodontitis; refractory chronic mucosal inflammation
Systemic dissemination & distant organ pathogenesis	Vascular endothelial cells, tumor cells, tissue-resident immune cells	FadA-mediated endothelial adhesion; transendothelial migration; modulation of distal tissue immune microenvironments; activation of pro-tumorigenic signaling	Hematogenous bacterial spread; secondary site colonization; suppression of anti-tumor immunity	Gastrointestinal malignancies; adverse pregnancy outcomes; atherosclerotic cardiovascular disease; neurodegenerative disorders

## 4 Diagnostic biomarker potential and therapeutic targeting of *Fusobacterium nucleatum*

### 4.1 Utility as a diagnostic and prognostic biomarker

Accumulating clinical evidence has validated F. nucleatum as a promising microbial biomarker for disease screening, early diagnosis and prognostic stratification across multiple pathological conditions. In colorectal cancer, fecal F. nucleatum abundance is significantly associated with tumor stage, histological grade and lymph node metastasis, and its combination with fecal immunochemical testing increases the diagnostic sensitivity for early-stage lesions by approximately 15%–20% compared with single-marker detection, offering a non-invasive adjunctive tool for population-based colorectal cancer screening (Zhang et al., 2019). In periodontal practice, the bacterial load in gingival crevicular fluid and saliva samples closely correlates with clinical attachment loss and probing depth, and can serve as a quantitative indicator to monitor disease activity and evaluate therapeutic response. Furthermore, high intratumoral F. nucleatum colonization is an independent prognostic factor for shorter overall survival and disease-free survival in various malignancies, and its predictive value for chemoresistance risk enables pretreatment risk stratification to guide individualized treatment decisions.

### 4.2 Therapeutic strategies targeting *F. nucleatum*

Diverse therapeutic approaches targeting F. nucleatum have been developed and evaluated in preclinical models, with some advancing to early clinical investigation. Adjuvant antibiotic therapy with metronidazole or clindamycin effectively reduces F. nucleatum burden in both oral and intestinal niches, and preclinical studies have demonstrated that antibiotic-mediated depletion of intratumoral F. nucleatum reverses 5-fluorouracil resistance and suppresses colorectal tumor progression (Chen et al., 2025). To avoid off-target disruption of commensal microbiota by broad-spectrum antibiotics, pathogen-specific interventions such as lytic bacteriophage therapy have emerged as a promising alternative. A novel F. nucleatum-specific lytic phage exhibits high species specificity and potent bactericidal activity, and selectively reduces tumor-associated bacterial load without altering the overall gut microbial community structure, resulting in significant tumor growth inhibition in mouse models (Lam et al., 2025). Additional investigational strategies include virulence factor inhibitors targeting FadA and Fap2 adhesins, and probiotic formulations that competitively block F. nucleatum colonization, both of which show favorable safety profiles and therapeutic potential in preclinical settings.

### 4.3 Challenges and future directions for clinical translation

Despite substantial preclinical progress, the clinical translation of F. nucleatum-based diagnostics and therapeutics remains hindered by several key challenges. First, the lack of standardized quantification protocols, uniform reference cutoff values and harmonized detection platforms across different clinical specimens has limited the reproducibility and comparability of study results, which is a critical barrier to routine clinical adoption (Quagliariello et al., 2025). Second, strain-level heterogeneity of F. nucleatum leads to divergent pathogenic potential and treatment susceptibility among different isolates, meaning that species-level detection alone is insufficient to guide precise clinical decision-making (Connolly and Kelly, 2025). Third, targeted delivery of therapeutic agents to deep colonization sites such as hypoxic tumor parenchyma and periodontal pockets remains a technical bottleneck that restricts *in vivo* bactericidal efficacy. Future research priorities include the development of point-of-care detection assays with standardized workflows, engineering of site-specific drug delivery systems, and exploration of combination regimens that integrate F. nucleatum-targeted therapy with cancer immunotherapy to achieve synergistic therapeutic benefits (Kowsarkhizi et al., 2025).

## 5 Microbial interaction networks and microecological regulatory effects

### 5.1 Interspecies co-aggregation and metabolic synergy in oral biofilms

F. nucleatum acts as a core intermediate colonizer in dental plaque biofilms with robust interspecies coaggregation activity verified in *in vitro* models. Through co-aggregation mediated by multiple surface adhesins, it forms physical connections between early-colonizing streptococci and late-colonizing anaerobic pathogens and serves as a structural scaffold in engineered *in vitro* biofilm systems (Kolenbrander et al., 2010). However, the structural bridging role of F. nucleatum in native *in vivo* biofilms remains controversial: ex vivo imaging analyses of human dental plaque samples indicate that F. nucleatum does not occupy a central hub position in the spatial architecture of native plaques, and biofilm assembly involves complex multilateral interactions rather than a strictly linear bridging pattern (Zhang et al., 2022). In addition, primary colonizer aggregates can pre-assemble in saliva prior to adhesion to the tooth surface, which bypasses the requirement for stepwise interspecies bridging on the salivary pellicle (Simon-Soro et al., 2022).

Its surface Fap2 protein recognizes galactose residues on streptococcal cell walls to form stable interspecies linkages, while RadD protein mediates specific binding to periodontal pathogens including Porphyromonas gingivalis and Aggregatibacter actinomycetemcomitans, facilitating their anchorage and proliferation in deep biofilm layers (Connolly and Kelly, 2025). At the metabolic level, short-chain fatty acids such as succinate and butyrate generated by amino acid fermentation of F. nucleatum serve as growth substrates for P. gingivalis and upregulate the expression of its cysteine proteases (Sakanaka et al., 2022; Leonov et al., 2023). Meanwhile, oxygen consumption by F. nucleatum depletes free oxygen in the local microenvironment, maintaining favorable growth conditions for obligate anaerobic pathogens. This dual function of structural scaffolding and metabolic support positions F. nucleatum as an important regulatory node that enhances the pathogenicity of periodontal biofilms in model systems.

### 5.2 Microbiota interactions in distal niches and cooperative disease progression

Following hematogenous dissemination to distal sites such as the intestinal tract and tumors, F. nucleatum interacts with local microbiota and collectively drives disease progression. In the colorectal mucosa, F. nucleatum disrupts epithelial tight junctions via its FadA adhesin and increases mucosal permeability, enabling normally luminal bacteria such as enterotoxigenic Escherichia coli and Bacteroides fragilis to invade the lamina propria and jointly induce chronic inflammation and abnormal tissue hyperplasia (Rubinstein et al., 2013). Within the tumor microenvironment, F. nucleatum-mediated immunosuppression exerts a synergistic effect with colibactin genotoxin produced by E. coli: the former suppresses anti-tumor immune responses, while the latter directly inflicts cellular DNA damage, and the two together accelerate malignant progression of tumor cells. In atherosclerotic plaques, co-colonization of F. nucleatum and P. gingivalis synergistically activates the NF-κB pathway, promotes secretion of inflammatory factors and matrix metalloproteinases by vascular smooth muscle cells, and elevates the degradation risk of the plaque fibrous cap (Yáñez et al., 2024).

### 5.3 Research progress and application limitations of microecological intervention strategies

For *F.* nucleatum-associated microbiota dysbiosis, microecological regulation as a supplement to antibiotic therapy has been evaluated in multiple preclinical and preliminary clinical studies. Regarding probiotics, multiple *Lactobacillus* strains inhibit adhesion and biofilm formation of *F. nucleatum* by competing for epithelial binding sites and secreting bacteriocin-like substances. *In vitro* experiments demonstrate that *Lactobacillus rhamnosus* GG reduces *F. nucleatum* biofilm biomass by 42% and significantly downregulates the expression of its virulence genes (Van Holm et al., 2023). Topical application of probiotic mouthwash in the oral cavity can lower *F. nucleatum* load in saliva and gingival crevicular fluid and help improve periodontal inflammatory indices, but microbiota rebound frequently occurs after treatment cessation. Fecal microbiota transplantation can reduce intestinal *F. nucleatum* abundance in some patients with ulcerative colitis, yet its adjuvant therapeutic value for colorectal cancer still lacks support from large-scale randomized controlled trials (Li et al., 2024). The core bottleneck of current microecological interventions is the difficulty in penetrating the extracellular matrix barrier of mature biofilms, plus large inter-individual variation in probiotic colonization efficiency at lesion sites. For these reasons, it cannot yet replace traditional targeted therapy and only serves as an auxiliary component of comprehensive prevention and control strategies (Saeed et al., 2022).

## 6 Frontier technological advances and clinical translation outlook

### 6.1 Multi-omics dissection of pathogen-host interactions

High-throughput multi-omics approaches have substantially deepened the mechanistic understanding of F. nucleatum-host crosstalk at strain-level resolution. Whole-genome sequencing paired with metagenomic profiling has identified distinct pathogenic lineages of F. nucleatum enriched in colorectal tumor tissues, which carry unique virulence gene clusters associated with epithelial invasion and intracellular survival that are absent in oral commensal isolates (Kostic et al., 2012). Single-cell RNA sequencing of patient tumor tissues further reveals that F. nucleatum colonization spatially correlates with exhausted CD8+ T cell subsets and M2-like tumor-associated macrophage polarization, and cell-cell interaction analysis pinpoints the CCR5/CCL5 axis as a key mediator of bacteria-driven immunosuppressive microenvironment formation (Ding et al., 2024). Metaproteomic and metabolomic profiling additionally shows that intratumoral F. nucleatum reprograms tumor cell amino acid metabolism and generates immunomodulatory metabolites that directly dampen dendritic cell antigen presentation capacity. These multi-layered omics data provide a systemic view of pathogen-host interactions and facilitate the identification of novel therapeutic targets.

### 6.2 Development of novel detection assays and point-of-care diagnostics

Traditional culture-based detection of F. nucleatum is limited by its strict anaerobic growth requirement and long turnaround time, driving the development of rapid, sensitive and field-deployable diagnostic technologies. Loop-mediated isothermal amplification (LAMP) assays targeting species-specific 16S rRNA gene sequences achieve detection limits comparable to quantitative PCR within 30 minutes, and can be performed with minimal laboratory equipment, making them suitable for primary care settings and resource-limited regions (Zuraik et al., 2024). Recent advances in CRISPR-Cas12a based detection systems further improve the sensitivity to single-cell level, and integrated microfluidic chips allow simultaneous quantification of multiple microbial biomarkers from a single clinical specimen, enabling rapid risk stratification of colorectal cancer in outpatient settings (Qu et al., 2024). Aptamer-based biosensors represent another emerging platform, enabling label-free, real-time detection of bacterial cells in saliva and fecal samples with high specificity against commensal microbiota. These technological advances lay the foundation for widespread clinical implementation of F. nucleatum-based screening and disease monitoring.

### 6.3 Preclinical validation and early clinical exploration of combination therapies

Emerging evidence supports that combining F. nucleatum-targeted interventions with standard cancer therapies yields synergistic therapeutic benefits, and several combination regimens have entered early phase clinical evaluation. Preclinical studies demonstrate that depletion of intratumoral F. nucleatum by targeted antibiotics not only reverses 5-fluorouracil chemoresistance but also enhances the efficacy of anti-PD-1 immunotherapy, by restoring intratumoral CD8+ T cell infiltration and reducing immunosuppressive myeloid cell populations (Jiang et al., 2023). Bacteriophage therapy in combination with chemotherapy shows improved tumor growth inhibition compared with monotherapy in mouse models, with lower risk of broad-spectrum antibiotic resistance and commensal microbiota disruption. Early phase clinical studies in recurrent colorectal cancer patients further indicate that adjuvant antibiotic treatment prior to anti-PD-1 therapy improves objective response rates in patients with high baseline F. nucleatum abundance, though large-scale randomized controlled trials are still required to confirm long-term survival benefits and optimal patient selection criteria (Gao et al., 2021).

## 7 Conclusion and future research perspectives

Fifteen years of accumulated research has firmly established F. nucleatum as a prototypical pathobiont that bridges oral dysbiosis and a spectrum of systemic disorders. Originating as a core structural species in periodontal biofilms, it disseminates via hematogenous and enteral routes to distant tissue niches, where it drives pathogenesis through evolutionarily conserved mechanisms: adhesion-mediated epithelial barrier breakdown, induction of an immunosuppressive microenvironment, reprogramming of host and tumor cell metabolism, and reshaping of local microbial networks to favor disease progression. These mechanistic insights have spurred the development of species-specific diagnostic assays and targeted therapeutic strategies, with multiple approaches showing encouraging preclinical activity and early clinical promise. Nonetheless, clinical translation faces substantial bottlenecks: extensive strain-level genetic and phenotypic diversity means findings from individual laboratory strains cannot be broadly generalized; technical limitations in *in vivo* tracking leave the temporal dynamics of colonization and disease initiation poorly characterized; most clinical evidence remains retrospective and associative rather than causal; and the long-term effects of targeted eradication on commensal microbial homeostasis and the risk of compensatory pathobiont overgrowth remain underexplored (Han, 2015; Zepeda-Rivera et al., 2024).

Looking ahead, large-scale pan-genomic and phenotypic profiling of clinical isolates across diverse populations and disease contexts is a foundational priority, as it will define functionally distinct virulence subtypes, identify robust biomarkers for patient risk stratification, and establish standardized experimental frameworks for cross-study comparison (Krieger et al., 2024). Advances in intravital imaging and single-cell spatial transcriptomics will further enable direct visualization of bacteria-host interactions at the cellular level, clarifying colonization dynamics and establishing causal links between pathogen infection and disease progression. Rigorously designed prospective randomized controlled trials are needed to confirm the adjuvant therapeutic benefits of F. nucleatum-targeted interventions in combination with standard treatments, and to define optimal dosing regimens, treatment timing, and patient selection criteria. Parallel development of next-generation delivery platforms, including bacteriophage cocktails and bacteria-specific nanoparticle carriers, will improve targeting precision and minimize off-target disruption to commensal microbiota (Wu et al., 2025). What is more noteworthy is that the current understanding of the biology of F. nucleatum still has significant limitations. Most of the evidence supporting its structural bridging function comes from *in vitro* co-culture systems, while the *in vivo* validation of its role in natural oral biofilms is still insufficient. The discovery of live biopsy plaque imaging based on fluorescence *in situ* hybridization (FISH) and the finding of pre-assembled bacterial aggregates in saliva have challenged the traditional stepwise bridging model, highlighting the complexity of the natural biofilm formation process. Future studies need to conduct *in situ* research with high-resolution spatial imaging to clarify the actual contribution of F. nucleatum to the structural organization of human oral biofilms. Integrating oral pathogen screening into routine health examinations may also support early risk identification and primary prevention, extending the public health impact of this research beyond therapeutic applications.

## References

[B1] Britton T. A. Lee J. H. Chang C. Bhat A. H. Chen Y.-W. Mohammed Ali R. . (2025). Inactivation of the Fusobacterium nucleatum Rnf complex reduces FadA-mediated amyloid formation and tumor development. mBio 16 (6), e0103225. doi: 10.1128/mbio.01032-25 40401912 PMC12153266

[B2] Chen F. Guo S. Li Y. Lu Y. Liu L. Chen S. . (2025). Fusobacterium nucleatum-driven CX3CR1+ PD-L1+ phagocytes route to tumor tissues and reshape tumor microenvironment. Gut Microbes 17 (1), 2442037. doi: 10.1080/19490976.2024.2442037 PMC12931713 39710592

[B3] Chen G. Sun Q. Cai Q. Zhou H. (2022). Outer membrane vesicles from Fusobacterium nucleatum switch M0-like macrophages toward the M1 phenotype to destroy periodontal tissues in mice. Front. Microbiol. 13, 815638. doi: 10.3389/fmicb.2022.815638 35391731 PMC8981991

[B4] Connolly J. P. Kelly L. (2025). The physical biogeography of Fusobacterium nucleatum in health and disease. mBio 16, e0298924. doi: 10.1128/mbio.02989-24 40062772 PMC11980382

[B5] Darveau R. P. (2010). Periodontitis: a polymicrobial disruption of host homeostasis. Nat. Rev. Microbiol. 8, 481–490. doi: 10.1038/nrmicro2337 20514045

[B6] Ding T. Chen Q. Liu H. Zhang H. Sun Y. Zhao L. . (2024). Single-cell RNA sequencing analysis reveals the distinct features of colorectal cancer with or without Fusobacterium nucleatum infection in PD-L1 blockade therapy. Heliyon 10 (18), e37511. doi: 10.1016/j.heliyon.2024.e37511 PMC11416490 39309908

[B7] Ellett F. Kacamak N. I. Alvarez C. R. Oliveira E. H. S. Hasturk H. Paster B. J. . (2023). Fusobacterium nucleatum dissemination by neutrophils. J. Oral Microbiol. 15 (1), 2217067. doi: 10.1080/20002297.2023.2217067 PMC10240972 37283724

[B8] Fan Z. Tang P. Li C. Yang Q. Xu Y. Su C. . (2022). Fusobacterium nucleatum and its associated systemic diseases: epidemiologic studies and possible mechanisms. J. Oral. Microbiol. 15 (1), 2145729. doi: 10.1080/20002297.2022.2145729 PMC9673791 36407281

[B9] Fardini Y. Wang X. Témoin S. Nithianantham S. Lee D. Shoham M. . (2011). Fusobacterium nucleatum adhesin FadA binds vascular endothelial cadherin and alters endothelial integrity. Mol. Microbiol. 82 (6), 1468–1480. doi: 10.1111/j.1365-2958.2011.07905.x PMC3237733 22040113

[B10] Farrugia C. Stafford G. P. Gains A. F. Cutts A. R. Murdoch C. (2022). Fusobacterium nucleatum mediates endothelial damage and increased permeability following single species and polymicrobial infection. J. Periodontol. 93, 1421–1433. doi: 10.1002/JPER.21-0671 35644006 PMC9796848

[B11] Gao Y. Bi D. Xie R. Li M. Guo J. Liu H. . (2021). Fusobacterium nucleatum enhances the efficacy of PD-L1 blockade in colorectal cancer. Signal Transduct Target Ther. 6 (1), 398. doi: 10.1038/s41392-021-00795-x PMC8602417 34795206

[B12] G C B Wu C. (2025). The CarSR two-component system directly controls radD expression as a global regulator that senses bacterial coaggregation in Fusobacterium nucleatum. J. Bacteriol. 207, e0052924. doi: 10.1128/jb.00529-24 40396725 PMC12186487

[B13] Griffen A. L. Beall C. J. Campbell J. H. Firestone N. D. Kumar P. S. Yang Z. K. . (2012). Distinct and complex bacterial profiles in human periodontitis and health revealed by 16S pyrosequencing. ISME J. 6 (6), 1176–1185. doi: 10.1038/ismej.2011.191 PMC3358035 22170420

[B14] Hajishengallis G. Lamont R. J. (2012). Beyond the red complex and into more complexity: the polymicrobial synergy and dysbiosis (PSD) model of periodontal disease etiology. Mol. Oral. Microbiol. 27, 409–419. doi: 10.1111/j.2041-1014.2012.00663.x 23134607 PMC3653317

[B15] Han Y. W. (2015). Fusobacterium nucleatum: a commensal-turned pathogen. Curr. Opin. Microbiol. 23, 141–147. doi: 10.1016/j.mib.2014.11.013 25576662 PMC4323942

[B16] Han Y. W. Ikegami A. Rajanna C. Kawsar H. I. Zhou Y. Li M. . (2005). Identification and characterization of a novel adhesin unique to oral fusobacteria. J. Bacteriol. 187 (15), 5330–5340. doi: 10.1128/JB.187.15.5330-5340.2005 PMC1196005 16030227

[B17] Jiang S. S. Chen Y. X. Fang J. Y. (2026). Fusobacterium nucleatum: ecology, pathogenesis and clinical implications. Nat. Rev. Microbiol. 24, 197–214. doi: 10.1038/s41579-025-01237-z 40983729

[B18] Jiang S. S. Xie Y. L. Xiao X. Y. Kang Z. R. Lin X. L. Zhang L. . (2023). Fusobacterium nucleatum-derived succinic acid induces tumor resistance to immunotherapy in colorectal cancer. Cell Host Microbe 31 (5), 781–797.e9. doi: 10.1016/j.chom.2023.04.010 37130518

[B19] Kaplan C. W. Lux R. Haake S. K. Shi W. (2009). The Fusobacterium nucleatum outer membrane protein RadD is an arginine-inhibitable adhesin required for inter-species adherence and the structured architecture of multispecies biofilm. Mol. Microbiol. 71, 35–47. doi: 10.1111/j.1365-2958.2008.06503.x 19007407 PMC2741168

[B20] Kikuchi Y. Kokubu E. Ishihara K. (2025). Fusobacterium nucleatum increases growth of motile bacterium Treponema denticola without contact *in vitro*. Bull. Tokyo Dent. Coll. 66, 145–156. doi: 10.2209/tdcpublication.2025-0015 41242734

[B21] Kolenbrander P. E. Palmer R. J. Periasamy S. Jakubovics N. S. (2010). Oral multispecies biofilm development and the key role of cell-cell distance. Nat. Rev. Microbiol. 8, 471–480. doi: 10.1038/nrmicro2381 20514044

[B22] Kostic A. D. Gevers D. Pedamallu C. S. Michaud M. Duke F. Earl A. M. . (2012). Genomic analysis identifies association of Fusobacterium with colorectal carcinoma. Genome Res. 22 (2), 292–298. doi: 10.1101/gr.126573.111 PMC3266036 22009990

[B23] Kowsarkhizi A. S. Yousefi B. Rahimi A. Aliramezani A. (2025). Targeting Fusobacterium nucleatum in colorectal cancer: therapeutic strategies and future directions. Infect. Agent Cancer 20, 70. doi: 10.1186/s13027-025-00676-w 41088139 PMC12522427

[B24] Krieger M. AbdelRahman Y. M. Choi D. Palmer E. A. Yoo A. McGuire S. . (2024). Stratification of Fusobacterium nucleatum by local health status in the oral cavity defines its subspecies disease association. Cell Host Microbe 32 (4), 479–488.e4. doi: 10.1016/j.chom.2024.02.010 PMC11018276 38479393

[B25] Kurgan Ş. Kansal S. Nguyen D. Stephens D. Koroneos Y. Hasturk H. . (2017). Strain-Specific Impact of Fusobacterium nucleatum on Neutrophil Function. J. Periodontol. 88 (4), 380–389. doi: 10.1902/jop.2016.160212 27762731

[B26] Lam H. Y. P. Lai M. J. Wang P. C. Wu W. J. Chen L. K. Fan H. W. . (2025). A Novel Bacteriophage with the Potential to Inhibit Fusobacterium nucleatum-Induced Proliferation of Colorectal Cancer Cells. Antibiotics (Basel) 14 (1), 45. doi: 10.3390/antibiotics14010045 PMC11760851 39858331

[B27] Leonov G. E. Varaeva Y. R. Livantsova E. N. Starodubova A. V. (2023). The complicated relationship of short-chain fatty acids and oral microbiome: a narrative review. Biomedicines 11, 2749. doi: 10.3390/biomedicines11102749 37893122 PMC10604844

[B28] Li D. H. Li Z. W. Sun Q. Wang L. Ning S. B. (2024). Lower fecal microbiota transplantation ameliorates ulcerative colitis by eliminating oral-derived Fusobacterium nucleatum and virulence factor. Gut Pathog. 16, 42. doi: 10.1186/s13099-024-00633-9 39118149 PMC11311926

[B29] Li Q. Luo W. Xiao L. Xu X. Peng X. Cheng L. . (2025). Microbial manipulators: Fusobacterium nucleatum modulates the tumor immune microenvironment in colorectal cancer. J. Oral Microbiol. 17 (1), 2544169. doi: 10.1080/20002297.2025.2544169 PMC12326384 40771747

[B30] Liang M. Liu Y. Zhang Z. Yang H. Dai N. Zhang N. . (2022). Fusobacterium nucleatum induces MDSCs enrichment via activation the NLRP3 inflammosome in ESCC cells, leading to cisplatin resistance. Ann. Med. 54 (1), 989–1003. doi: 10.1080/07853890.2022.2061045 PMC9891225 35435776

[B31] Meng Q. Gao Q. Mehrazarin S. Tangwanichgapong K. Wang Y. Huang Y. . (2021). Fusobacterium nucleatum secretes amyloid-like FadA to enhance pathogenicity. EMBO Rep. 22 (7), e52891. doi: 10.15252/embr.202152891 PMC8406402 34184813

[B32] Park Y. Simionato M. R. Sekiya K. Murakami Y. James D. Chen W. . (2005). Short fimbriae of Porphyromonas gingivalis and their role in coadhesion with Streptococcus gordonii. Infect Immun. 73 (7), 3983–3989. doi: 10.1128/IAI.73.7.3983-3989.2005 PMC1168573 15972485

[B33] Peng X. Cheng L. You Y. Tang C. Ren B. Li Y. . (2022). Oral microbiota in human systematic diseases. Int J Oral Sci. 14 (1), 14. doi: 10.1038/s41368-022-00163-7 PMC8891310 35236828

[B34] Qu H. Zhang W. Li J. Fu Q. Li X. Wang M. . (2024). A rapid and sensitive CRISPR-Cas12a for the detection of Fusobacterium nucleatum. Microbiol Spectr. 12 (2), e0362923. doi: 10.1128/spectrum.03629-23 PMC10845955 38197659

[B35] Quagliariello V. Forte P. Ciappina G. Colarusso L. Giorgi C. Fiorica F. . (2025). Fusobacteriumnucleatum: Pathophysiological and Clinical Involvement in Inflammatory Bowel Diseases, Colorectal Cancer and Cardiovascular Diseases. Cancers (Basel) 17 (20), 3348. doi: 10.3390/cancers17203348 PMC12563274 41154402

[B36] Rubinstein M. R. Wang X. Liu W. Hao Y. Cai G. Han Y. W. (2013). Fusobacterium nucleatum promotes colorectal carcinogenesis by modulating E-cadherin/β-catenin signaling via its FadA adhesin. Cell Host Microbe 14, 195–212. doi: 10.1016/j.chom.2013.07.012 23954158 PMC3770529

[B37] Saeed M. Shoaib A. Kandimalla R. Javed S. Almatroudi A. Gupta R. . (2022). Microbe-based therapies for colorectal cancer: Advantages and limitations. Semin Cancer Biol. 86 (Pt 3), 652–665. doi: 10.1016/j.semcancer.2021.05.018 34020027

[B38] Sakanaka A. Kuboniwa M. Shimma S. Alghamdi S. A. Mayumi S. Lamont R. J. . (2022). Fusobacterium nucleatum Metabolically Integrates Commensals and Pathogens in Oral Biofilms. mSystems 7 (4), e0017022. doi: 10.1128/msystems.00170-22 PMC9426547 35852319

[B39] Simon-Soro A. Ren Z. Krom B. P. Hoogenkamp M. A. Cabello-Yeves P. J. Daniel S. G. . (2022). Polymicrobial Aggregates in Human Saliva Build the Oral Biofilm. mBio 13 (1), e0013122. doi: 10.1128/mbio.00131-22 PMC8903893 35189700

[B40] Van Holm W. Carvalho R. Delanghe L. Eilers T. Zayed N. Mermans F. . (2023). Antimicrobial potential of known and novel probiotics on *in vitro* periodontitis biofilms. NPJ Biofilms Microbiomes 9 (1), 3. doi: 10.1038/s41522-023-00370-y PMC9867767 36681674

[B41] Wu T. Zhang F. Liu H. Ma F. Yu Y. Sun D. . (2025). Fusobacterium nucleatum-targeted polymeric micelles disrupting biofilm-immune crosstalk for precision colorectal cancer immunotherapy. J Control Release 388 (Pt 2), 114400. doi: 10.1016/j.jconrel.2025.114400 41218696

[B42] Yamaguchi-Kuroda Y. Kikuchi Y. Kokubu E. Ishihara K. (2023). Porphyromonas gingivalis diffusible signaling molecules enhance Fusobacterium nucleatum biofilm formation via gene expression modulation. J. Oral. Microbiol. 15, 2165001. doi: 10.1080/20002297.2023.2165001 36687169 PMC9848294

[B43] Yáñez L. Soto C. Tapia H. Pacheco M. Tapia J. Osses G. . (2024). Co-Culture of P. gingivalis and F. nucleatum Synergistically Elevates IL-6 Expression via TLR4 Signaling in Oral Keratinocytes. Int J Mol Sci. 25 (7), 3611. doi: 10.3390/ijms25073611 PMC11011619 38612423

[B44] Yang X. Zhang S. Ning T. Wu J. (2025). Fusobacterium nucleatum in health and disease. MedComm 6, e70465. doi: 10.1002/mco2.70465 41216129 PMC12596987

[B45] Yu T. Guo F. Yu Y. Sun T. Ma D. Han J. . (2017). Fusobacterium nucleatum Promotes Chemoresistance to Colorectal Cancer by Modulating Autophagy. Cell. 170 (3), 548–563.e16. doi: 10.1016/j.cell.2017.07.008 PMC5767127 28753429

[B46] Zepeda-Rivera M. Minot S. S. Bouzek H. Wu H. Blanco-Míguez A. Manghi P. . (2024). A distinct Fusobacterium nucleatum clade dominates the colorectal cancer niche. Nature 628 (8007), 424–432. doi: 10.1038/s41586-024-07182-w PMC11006615 38509359

[B47] Zhang M. Whiteley M. Lewin G. R. (2022). Polymicrobial interactions of oral microbiota: a historical review and current perspective. mBio 13, e0023522. doi: 10.1128/mbio.00235-22 35491817 PMC9239150

[B48] Zhang X. Zhu X. Cao Y. Fang J. Y. Hong J. Chen H. (2019). Fecal Fusobacterium nucleatum for the diagnosis of colorectal tumor: a systematic review and meta-analysis. Cancer Med. 8, 480–491. doi: 10.1002/cam4.1850 30636375 PMC6382715

[B49] Zuraik A. A. Daboul Y. Awama M. A. Yazigi H. Kayasseh M. A. Georges M. (2024). Rapid detection of FadA in Fusobacterium nucleatum using the quantitative LAMP colorimetric phenol red method in stool samples from colorectal cancer patients. Sci. Rep. 14, 13739. doi: 10.1038/s41598-024-62846-x 38877111 PMC11178829

